# Selection of Reference Genes of Flower Development in *Ludisia discolor*

**DOI:** 10.3390/genes15091225

**Published:** 2024-09-19

**Authors:** Rui Gao, Wenyan He, Wen-Tao Zhu, Xuewei Zhao, Chen Chen, You Wu, Shasha Wu, Jun-Wen Zhai, Zhong-Jian Liu

**Affiliations:** 1 Key Laboratory of National Forestry and Grassland Administration for Orchid Conservation and Utilization at College of Landscape Architecture, Fujian Agriculture and Forestry University, Fuzhou 350002, China; 13961833825@163.com (R.G.); wenyan19982021@163.com (W.H.); rugosus2518@gmail.com (W.-T.Z.); zxw6681@163.com (X.Z.); 12319075004@fafu.edu.cn (C.C.); 15980280917@163.com (Y.W.); shashawu1984@126.com (S.W.); 2 College of Forestry, Fujian Agriculture and Forestry University, Fuzhou 350002, China

**Keywords:** *Ludisia discolor*, RT-qPCR, reference gene, stability evaluation

## Abstract

**Background:** RT-qPCR is a powerful strategy for recognizing the most appropriate reference genes, which can successfully minimize experimental mistakes through accurate normalization. *Ludisia discolor*, recognized for its ornamental value, features little, distinctive blossoms with twisted lips and gynostemium showing chiral asymmetry, together with striking blood-red fallen leaves periodically marked with golden blood vessels. **Methods and Results:** To ensure the accuracy of qRT-PCR, selecting appropriate reference genes for quantifying target gene expression levels is essential. This study aims to identify stable reference genes during the development of *L. discolor*. In this study, the entire floral buds, including the lips and gynostemium from different development stages, were taken as materials. Based upon the transcriptome information of *L. discolor*, nine housekeeping genes, *ACT*, *HIS*, *EF1-α1*, *EF1-α2*, *PP2A*, *UBQ1*, *UBQ2*, *UBQ3*, and *TUB*, were selected in this research study as prospect interior referral genes. The expression of these nine genes were found by RT-qPCR and afterwards comprehensively examined by four software options: geNorm, NormFinder, BestKeeper, and ΔCt. The outcomes of the analysis showed that *ACT* was the most steady gene, which could be the most effective inner referral gene for the expression evaluation of flower advancement in *L. discolor*. **Conclusions:** The results of this study will contribute to the molecular biology research of flower development in *L. discolor* and closely related species.

## 1. Introduction

Real-time quantitative PCR (RT-qPCR) is renowned for its high specificity, broad dynamic variety, and cost-effectiveness. This commonly utilized technique for measuring genetics expression has been thoroughly utilized in verifying gene expression levels [1]. Calculating the family member expression of genetics utilizing RT-qPCR demands introducing reference genetics with fairly steady expression for information normalization. This step is crucial for getting rid of variations and operational mistakes between various samples [2,3,4]. Referral genetics, additionally referred to as housekeeping genetics, constantly displays stable expression throughout various cell kinds and physical problems. Instances include actin (*ACT*), β-tubulin (*TUB*), elongation factor 1-α (*EF1-α*), and polyubiquitin (*UBQ*). Nevertheless, these generally utilized referral genes do not constantly show steady expression under all scenarios. Research suggests that one of the most stable referral genes can differ amongst different types, and genes that are fairly steady under specific speculative problems may also be different [5,6,7,8]. For that reason, picking ideal recommendation genetics is important for certain varieties and speculative problems.

*L. discolor* (*Ludisia*, Orchidaceae) is a perennial lithophytic herb. It is native to southern China and tropical Asia [9]. This plant is distinguished by its small and unique flowers, which exhibit twisted, chiral asymmetry in the lip and gynandrium. The entire flower is white with a yellow gynandrium, making it highly prized for ornamental purposes. *L. discolor* possesses significant medicinal properties, as the entire plant can be used therapeutically. Due to its unique morphological features, *L. discolor* is also highly valuable in plant developmental biology, particularly in research focused on pigment biosynthesis and floral development. Currently, there have been few research studies on the molecular systems underlying flower pigmentation and chiral crookedness in *L. discolor*. Using molecular techniques to uncover vital genetics related to blossom advancement is essential for advancing the molecular reproduction of *L. discolor*.

*MYB* transcription variables are critical in the growth of flowers, influencing the morphology, shade, and body organ identification of flower frameworks. In *Arabidopsis thaliana*, *MYB21* [10] and *MYB24* [11] are involved in stamen development, significantly impacting stamen reproductive capacity. In *Antirrhinum majus* [12], *MYB* transcription factors regulate floral organ development, affecting floral symmetry. Despite their known roles, research on flower development in *L. discolor* remains limited. Transcriptomic analyses of *L. discolor* have revealed that *MYB* transcription factors are implicated in the chiral asymmetric development of floral organs. Consequently, we identified four genes with significantly differential expression and assessed their expression accounts using RT-qPCR. Nevertheless, due to the lack of research studies on recommendation genetics in *L. discolor*, precise metrology of genetics expression using RT-qPCR continues to be tough.

To accurately screen for reference genes, this study selected nine genes that have been validated in other species as candidate reference genes, and primers were designed accordingly. Utilizing transcriptome data from the lip and gynandrium of *L. discolor* at four developmental phases, and utilizing RT-qPCR innovation, the amplification efficiency and cycle limit (Ct) values of these nine referral genes were assessed across numerous flower body organs at four developmental phases, and the expression of prospect genetics in blossom organs was analyzed. The security of these genetics was evaluated, making use of GeNorm [13], NormFinder [14], BestKeeper [15], and ΔCt [16]. The most secure genetics was identified and verified for stability using four target genes connected to flower advancement, therefore identifying the most suitable reference gene. The aim of this study is to provide a foundation for subsequent gene expression analyses and it serves as a reference for the selection of reference genes in closely related species.

## 2. Materials and Methods

### 2.1. Plant Materials

**Figure 1:**
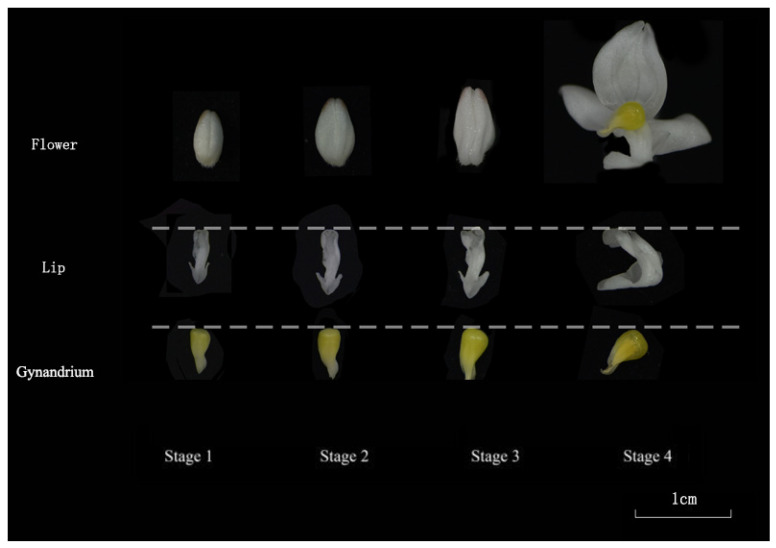
*L. discolor* flower of four development stages. S1 (small-bud stage), S2 (medium-bud stage), S3 (large-bud stage), and S4 (full-bloom stage).

Materials for this experiment consisted of *L. discolor* samplings provided from the greenhouse at the Forest Orchid Garden of Fujian Agriculture and Forestry University, Fuzhou, China. Examples were accumulated from the labellum and gynandrium at 4 developmental stages, S1 (small-bud stage), S2 (medium-bud stage), S3 (large-bud stage), and S4 (full-bloom stage), resulting in eight distinct samples (Figure 1). Each example included three organic duplicates. Following collection, the samples were quickly put in liquid nitrogen and saved at −80 °C.

### 2.2. Total RNA Isolation and cDNA Synthesis

Total RNA was removed from the lip and gynandrium of *L. discolor* at four distinct developmental stages using OMEGA Plant Total RNA Extraction Kit (Magen, Guangzhou, China), adhering to the maker’s guidelines. The top quality of the drawn-out overall RNA was analyzed via 1% agarose gel electrophoresis, while the concentration and purity were figured out utilizing a NanoDrop UV-Vis spectrophotometer (Thermo, Waltham, MA, USA). RNA was reverse-transcribed right into cDNA using the All-in-One First-Strand SuperMix kit (Magen, Guangzhou, China), following the manufacturer’s method. The resulting cDNA was stored at −20 °C for the succeeding analysis.

### 2.3. Selection of Candidate Reference Genes and Primer Design

Based upon the transcriptome data of *L. discolor*’s labellum and gynandrium at various blooming stages (unpublished), referral genes were drawn out as referral series from *A. thaliana* and *Phalaenopsis* [17]. TBLASTn was carried out within the *L. discolor* transcriptome to select genetics with high FPKM values and very little differential expression throughout samples. A total amount of 9 recommendation genes were determined. RT-qPCR guides were created based on the nucleotide sequences of the prospect genetics, making use of the Primer3 online device (Table 1). The primers had a Tm value of 59–61 °C, a GC material of 45–50%, a guide length of 20–22 bp, and a product length of 106–131 bp. All guides were manufactured by Sangon Biotech (Shanghai) Co., Ltd. (Shanghai, China).

### 2.4. RT-qPCR and Amplification Efficiency

After equal amounts of cDNA templates from different developmental stages and floral tissues were mixed, five serial dilutions were prepared for RT-qPCR, with each reaction performed in quadruplicate. The Ct values of the reference genes across the five dilution gradients were plotted on the y-axis, while the logarithm of the dilution factor was plotted on the x-axis. A standard curve was generated using Microsoft Excel, and amplification efficiency for each candidate reference gene was calculated using the formula E = (10^−1/slope^ − 1) × 100%. The goodness-of-fit of the regression equation was determined by the R^2^ value to evaluate the reliability of the standard curve [18]. Each reaction was conducted in triplicate. The RT-qPCR actions were performed following the instructions of the Hieff qPCR SYBR Green Master Mix (Low Rox Plus) kit from Yeasen (Yeasen, Shanghai, China). The 20 µL reaction system included 10 µL of Hieff qPCR SYBR Green Master Mix (Low Rox Plus), 0.4 µL of a forward primer (10 µM), 0.4 µL of a reverse primer (10 µM), 2 µL of cDNA, and 7.2 µL of dd H_2_O. The reactions were conducted on a 96-well plate. The reactions were conducted within a Thermo QuantStudio 1 Plus quantitative PCR instrument (Thermo, Waltham, MA, USA) and the amplification program was as follows: initial denaturation at 95 °C for 5 min, followed by 40 cycles of denaturation at 95 °C for 10 s and annealing at 60 °C for 30 s. A melting curve evaluation was performed to identify the specificity of the primers.

### 2.5. Data Analysis and Validation of Selected Candidate Reference Genes

Following the analytical methods from existing studies on reference gene stability, Microsoft Excel 2021 was used to process and analyze the Ct values obtained in the experiment. These processed Ct values were then used directly for ΔCt and BestKeeper analyses. The raw Ct values were converted into relative variables by Livak, and the formula E = Q^−ΔCt^ was applied in Genorm and NormFinder analyses to calculate stability values, where E represents the amplification efficiency [19]. Finally, the most appropriate reference gene was identified through a comprehensive stability ranking across the software platforms.

To validate the reliability and accuracy of the selected reference genes, four members of the *MYB* gene family were chosen from the transcriptome based on their expression patterns related to blossom color development in *L. discolor* (Table 2). Their expression levels were quantitatively examined, making use of RT-qPCR, with each example analyzed in triplicate. The most steady and the very least stable referral genetics determined were utilized to examine the expression of the target genetics.

## 3. Results and Analysis

### 3.1. Primer Specificity Testing and PCR Amplification Efficiency

**Figure 2:**
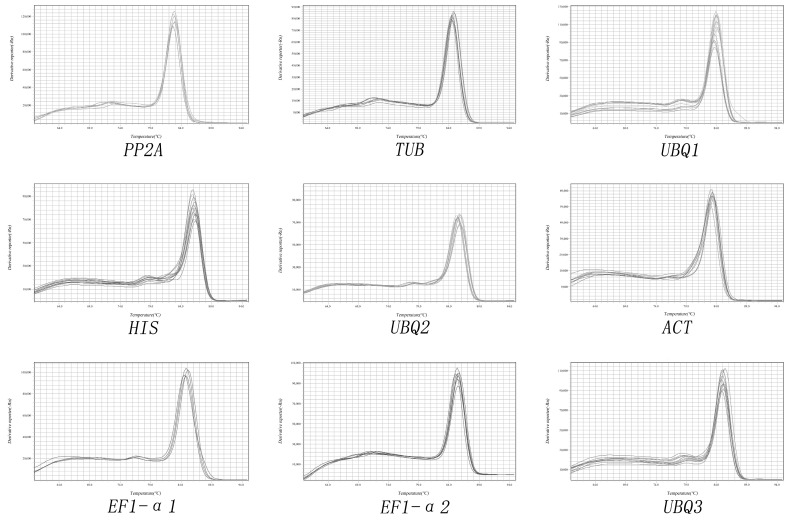
Melting curves of nine candidate RGs. Melting temperatures were visualized by plotting thenegative first derivative of fluorescence relative to the temperature in Celsius.

The real-time fluorescence quantitative analysis disclosed that the melt contours of each candidate referral gene primer showed single heights (Figure 2), showing the lack of guide dimers. This validates the high uniqueness of the designed guides, thus verifying the experimental information for subsequent analyses. The amplification efficiency analysis for the candidate reference genes reveals that all genes exhibit efficiencies exceeding 90%, with *EF1-α1*, *ACT*, and *UBQ1* displaying efficiencies greater than 100%. The correlation coefficients of these genes range from 0.981 to 0.999, with the exception of *UBQ1* and *UBQ2*, whose coefficients are below 0.99. These findings indicate that most reference genes demonstrate robust amplification efficiency (Table 3).

### 3.2. Analysis of Expression Levels of Candidate Reference Genes

**Figure 3:**
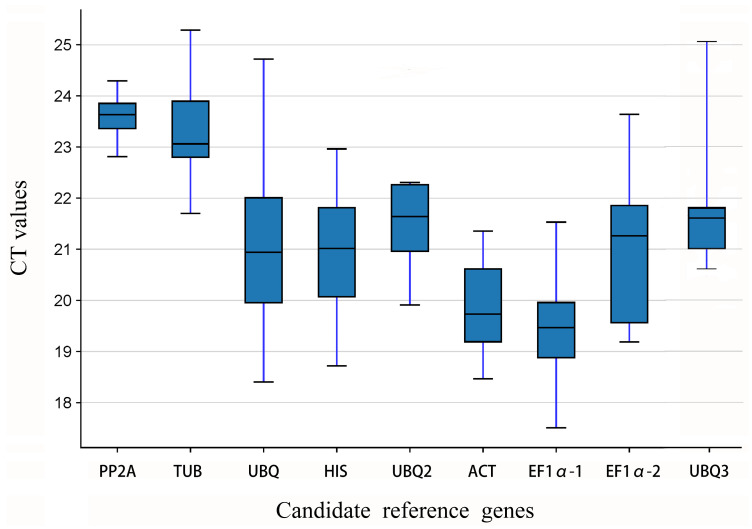
RT-qPCR CT values for all candidate reference genes in all samples.

Ct values are used to examine gene expression levels in RT-qPCR. A greater Ct worth for candidate referral genetics indicates reduced expression degrees, whereas a reduced Ct worth suggests higher expression degrees. Greater change in Ct values for the very same genetics shows reduced stability, while lower fluctuation shows greater security. The Ct values of nine prospect referral genes were examined throughout numerous samples, ranging from 19 to 25, during four stages of blossom development in *L. discolor*. Among these, *EF1-α1* had the smallest Ct values at 19.38, showing the greatest ordinary expression degree. *PP2A* had the greatest ordinary Ct values at 23.63, suggesting the lowest typical expression level. Figure 3 shows that the Ct values for *PP2A* are the most concentrated, whereas those for *EF1-α2* exhibit the greatest dispersion. *UBQ* has the largest difference between its maximum and minimum Ct values, while *PP2A* displays the smallest variation. The Ct values for *PP2A* and *TUB* are relatively high, indicating lower expression levels, while *EF1-α2* and *ACT* show lower Ct values, corresponding to higher expression levels.

### 3.3. Analysis of Expression Stability of Candidate Reference Genes

#### 3.3.1. GeNorm Analysis

Gene stability values (M) of candidate reference genes were established utilizing geNorm. A lower M value indicates higher stability in genetics expression, with values below 1.5 thought to be optimal for recommendation genes. Results indicated that all nine candidate referral genes displayed M values listed below 1.5 throughout numerous periods, indicating durable security [18]. The positions were in adherence to *ACT* = *HIS* > *EF1-α1* > *TUB* > *EF1-α2* > *UBQ1* > *PP2A* > *UBQ3* > *UBQ2*. *ACT* and *HIS* were identified as the most stable with an M value of 0.55, while *UBQ2* exhibited the highest M value at 1.34 (Table 4).

#### 3.3.2. NormFinder Analysis

The stability analysis and position of nine prospect referral genes were executed, making use of NormFinder. Reduced security values suggest even more consistent gene expression degrees. According to Table 5, *EF1-α1* demonstrated the greatest expression security, with a worth of 0.506, while *UBQ2* displayed the lowest security, with a value of 1.459.

#### 3.3.3. BestKeeper Analysis

BestKeeper ranks interior recommendation genetics based on their standard deviation (SD) and coefficient of variation (CV). Genes with lower SD and CV values are considered more stable, whereas those with SD > 1.0 are deemed unsuitable as reference genes [15]. As displayed in Table 6, *HIS*, *EF1-α2*, and *UBQ1* had SD values going beyond 1, suggesting poor stability and unsuitability as reference genes. *PP2A* was identified as the most secure, while *UBQ2* displayed lower stability.

#### 3.3.4. ΔCt Method

The ΔCt algorithm ranks gene expression stability based on the standard deviation, with lower values indicating higher stability. As shown in Table 6, the nine candidate reference genes are ranked in descending order of stability: *EF1-α1* > *ACT* > *HIS* > *TUB* > *EF1-α2* > *UBQ1* > *PP2A* > *UBQ3* > *UBQ2*. Both the ΔCt and NormFinder algorithms consistently identify *EF1-α1* as the most stable gene, with an STDEV value of 1.09. The rankings for *EF1-α1*, *ACT*, *HIS*, *TUB*, and *EF1-α2* are identical between the ΔCt and NormFinder analyses. Conversely, *UBQ2* shows the lowest expression stability, as indicated by both NormFinder and geNorm, with an STDEV value of 1.69 (Table 7).

#### 3.3.5. Comprehensive Analysis

Both similarities and differences were observed across all analysis results. To integrate the findings, the geometric mean of the stability positions from the three software program assessments was determined. A smaller geometric mean indicates greater gene stability. According to the geNorm analysis, *ACT* and *HIS* exhibited the highest stability. The NormFinder analysis identified *EF1-α1* as the most stable, whereas the BestKeeper analysis indicated that *PP2A* had the highest stability. The geometric mean of these results was calculated (Table 8). The findings indicated that *ACT* exhibited the highest stability across various stages of flower development in *L. discolor*, making it the most suitable reference gene. *EF1-α1* was the next most stable, whereas *UBQ2* was the least stable and unsuitable as a reference gene.

### 3.4. Reference Gene Validation

**Figure 4:**
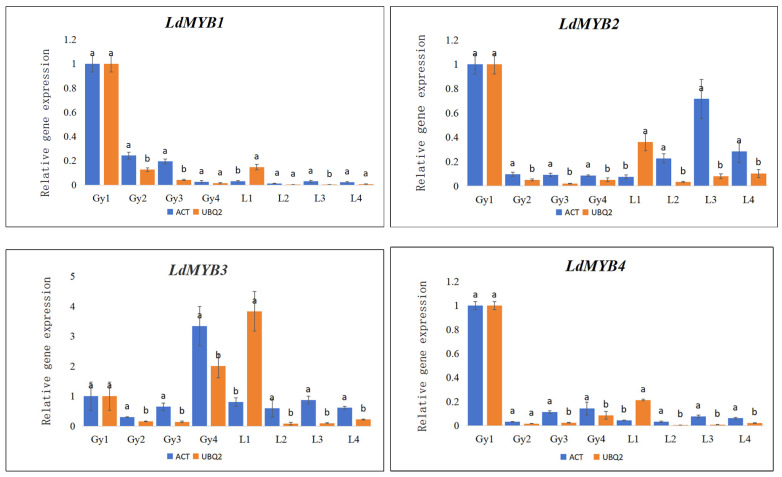
The RT-qPCR verification of four genes in different flower structures. ^a^ and ^b^ represent significant difference (*p* < 0.05). In each bar chart, the blue bars represent the best RGs (*ACT*), respectively. And the orange bars represent the worst RG (*UBQ2*). Gy1–4 represent the gynandrium of four floral organ developmental stages; L1–4 represent the lip of four floral organ developmental stages.

Based on the security analysis results, the relative expression levels of four *MYB*s associated with blossom color were determined, making use of both one of the most steady recommendation genes, *ACT*, and the least steady reference gene, *UBQ2*. Transcriptome data of *L. discolor* indicated that *LdMYB1*, *LdMYB2*, and *LdMYB4* show comparable expression patterns, with higher expression levels in the gynostemium during the S1 stage (Figure 4). The RT-qPCR recognition of the four target genes was performed using both the most stable reference gene, *ACT*, and the least stable referral gene, *UBQ2*. When *ACT* was utilized, the expression trends of the four target genes were consistent with the differential expression trends observed in the transcriptome data. In contrast, when the unsteady *UBQ2* was utilized for normalization, the relative expression levels of the four *MYB*s displayed partially aberrant trends.

For *LdMYB1*, the expression levels from L1 to L4 were comparable, regular with the pattern when *ACT* was used as the reference genetics. Nevertheless, when *UBQ2* was utilized as the reference genetics, the expression level at the L1 stage was dramatically higher than at the other phases. For *LdMYB2*, the expression degrees showed a progressive boost from L1 to L3. When *ACT* was used as the referral gene, the fad matched the transcriptome data, revealing an initial reduction followed by a rise. For *LdMYB3*, the expression level was highest at Gy4, and *ACT* matched the trend much better than *UBQ2*. For *LdMYB4*, the expression levels of L1 and L2 were similar, regular with the fad when *ACT* was made use of as the referral gene. Nonetheless, when *UBQ2* was made use of as the reference gene, there was a considerable decline in expression from L1 to L2.

Although in some samples, such as throughout the Gy1 to Gy4, the expression patterns of the prospect genetics were normally constant when utilizing either reference genetics, generally, *UBQ2* was less reliable than *ACT* for normalizing the family member expression degrees of the prospect genetics. In contrast, *ACT* more precisely mirrored the expression patterns of the target genetics, making it the ideal reference gene.

## 4. Discussion

RT-qPCR and transcriptome sequencing are crucial for elucidating gene expression dynamics during plant growth and development, especially for understanding the diverse roles of genes in flowers [20]. However, using reference genes from other species without validation can result in experimental errors. Therefore, introducing suitable species-specific reference genes is essential for obtaining accurate results. Extensive studies have been conducted to select suitable reference genes in various ornamental flowers, including *Chrysanthemum morifolium*, *Paeonia lactiflora*, and *Jasminum sambac* [21]. In the orchid family, studies have reported reference genes in species such as *Cymbidium ensifolium*, *Dendrobium*, and *Phalaenopsis* [18,22,23]. However, suitable recommendation genetics for *L. discolor* has not actually been reported. This research represents the first effort to display and verify referral genetics for RT-qPCR normalization in the organs of *L. discolor*, thus supplying crucial theoretical support for gene expression evaluation.

The Ct values of the candidate reference genes were evaluated using three software devices: geNorm, NormFinder, and BestKeeper. Due to differences in their algorithms, the evaluations of reference gene stability by these tools showed some discrepancies [24]. GeNorm selects two stable reference genes and their optimal combination in experimental samples based on the Vn/Vn + 1 value. This method effectively corrects systematic biases, resulting in more accurate assessments of gene expression stability. NormFinder operates similarly to geNorm. However, it identifies only one optimal reference gene at a time and cannot determine the best combination of reference genes. BestKeeper can analyze the variation in multiple sets of experimental reference genes, though it is limited to analyzing between three and ten genes. However, because of distinctions in calculation concepts among the three software applications, evaluations of the same example using these programs may generate varying study results. In the thorough evaluation, the results obtained from geNorm and NormFinder revealed a high degree of consistency, both suggesting that *ACT* displayed high stability. Nevertheless, the rise from BestKeeper differed dramatically from those of geNorm and NormFinder. Despite these distinctions, *UBQ2* continually rated as reduced in all three software program analyses, suggesting its inadequate security as a reference gene. In the study of reference genetics in numerous tissues of *J. sambac* [21], *ACT* revealed the least security in BestKeeper, while demonstrating higher security in geNorm and NormFinder [25]. As a result, the geometric mean of the results from all three software application devices was computed to identify the most secure and excellent reference gene. In this research, the geometric mean of the security values from geNorm, NormFinder, and BestKeeper was determined. Based on the results, *ACT* was figured out to have the highest stability, while *UBQ2* had the most affordable stability.

In research studies of flower growth, *MYB* is the biggest family of transcription factors (TFs) in plants [26]. *MYB* transcription elements typically contain several incomplete *MYB* repeat sequences (R), which with each other form the *MYB* DNA-binding domain name [27]. Among all *MYB* kinds, the *R2R3-MYB*, which has two R sequences, plays a leading and main duty in controlling anthocyanin biosynthesis paths (ABPs) [1,28]. *MYB* transcription aspects are associated with controlling numerous additional metabolic paths, consisting of tissue advancement, disease resistance, signal transduction, and flavonoid biosynthesis. Nonetheless, there are few reports on these features in *L. discolor*.

To validate the efficiency and reliability of the recommendation genes recognized in *L. discolor*, four key *MYB* genes involved in flower advancement were made use of as target genes to assess their expression patterns during the blossom advancement procedure. When *ACT* was used as the referral gene, the expression fads of *MYB*s were consistent with the transcriptome data, and all four *MYB*s displayed significant differential expression. On the other hand, when *UBQ2* was utilized as the recommendation genetics, the expression patterns of *MYB*s were inconsistent with the transcriptome information, indicating that using an unstable referral gene can bring about inaccurate differential expression outcomes. This recognition highlights the crucial significance of choosing a suitable reference gene to stay clear of substantial variances in outcomes. In this research study, *ACT* was determined as the most appropriate referral genetics for a blossom development research study in *L. discolor*.

In an ideal scenario, an optimal internal reference gene should exhibit stable expression regardless of cell type, developmental stage, or different treatment methods. However, there is no single ideal internal reference gene that applies to all plants. *ACT*, a structural protein, is integral to numerous cellular biological processes, including cell division, differentiation, polar transport, and signal transduction [29]. Due to its consistent expression across various tissues throughout all developmental stages and minimal variation, it is frequently utilized as an internal reference gene. Specifically, *ACT* has been identified as the most suitable internal reference gene in the pistils of *Brassica oleracea* at different stages. In diverse floral organs of *Phalaenopsis aphrodite*, including species such as *Cymbidium goeringii* and *Dendrobium chrysotoxum*, *ACT* also serves as an ideal internal reference gene across various developmental stages [18,30]. Additionally, *ACT* demonstrates stable expression in *Sorghum sudanense* (Piper) Stapf and exhibits the highest stability in leaf tissues of wax gourd under high-temperature stress conditions [31,32].

*UBQ*, a gene associated with healthy protein destruction, has been thought to be the most appropriate interior recommendation genetics in various tissues of *P. lactiflora* [33], as well as for measurable studies of dry spell stress-related genetics in *Trifolium repens* [34]. Nevertheless, in this research, *UBQ* displayed the poorest expression stability. On the other hand, *ACT* exhibited the highest security in this study, although it was found to be unsteady in *Lagerstroemia indica* [30,35] and had comparable findings in *P. aphrodite* [21]. *PP2A* exhibited intermediate security in this research; however, it has actually been recognized as one of the most suitable inner reference genes in studies including different selections of *Abelmoschus esculentus* [17]. *EF1-α* was determined as one of the most ideal interior referral genes in the expression analysis of stress-responsive genetics in *Chenopodium album* [36]. However, in this research study, its security rated was similar to findings in *Liriodendron hybrids* [37]. In *Citrus reticulata* [38], *GAPDH* exhibits the greatest expression security under drought anxiety, whereas *ACT* is the most steady under low-temperature anxiety. In a similar way, in *Lycopersicon esculentum* under abiotic stress and anxiety [39], *EF1-α* maintains stable expression under low-temperature problems, unlike *GAPDH*, which shows bad stability. Under low-temperature stress in the leaves of *Phyllostachys eduli*, *EF1-α* is one of the most stable, followed by *ACT*, and lastly *GAPDH*. In contrast, under high-temperature anxiety in *Solanum melongena*, *EF1-α* is the most steady prospect reference gene [40]. A research study examining the option of reference genetics for RT-qPCR in *Zea mays* under hormone treatment conditions recognized *EF1-α*, *TUB*, and *GAPDH* as the most appropriate recommendation genes under ABA hormonal agent stress and anxiety treatment. These results show that various plants, various components, or various therapies result in differing internal recommendation genetics. No solitary group of genetics is widely relevant across all plants. When inner referral genetics show substantial expression fluctuations, it ends up being tough to accurately calculate the expression patterns of target genes. Therefore, it is important to pick one of the most proper inner referral genes based on the speculative materials and treatments, enabling a quantitative analysis of target genes via RT-qPCR. This study stands for the initial effort to recognize interior recommendation genetics in *L. discolor*, verified together with genes associated with blossom growth. *ACT* was determined as one of the most suitable inner referral genes, offering an ideal referral for subsequent genetics expression evaluation. Additionally, it serves as a recommendation for picking internal reference genetics in very closely related types.

## 5. Conclusions

This study reviewed the stability of nine candidate reference genes connected to the growth of *L. discolor* utilizing RT-qPCR, examined with geNorm, NormFinder, and BestKeeper. The overall security ranking was identified as complying with *ACT* > *EF1-α1* > *HIS* > *PP2A* > *TUB* > *EF1-α2* > *UBQ3* > *UBQ1* > *UBQ2*, with *ACT* showing the highest possible stability and being one of the most ideal referral genetics, while *UBQ2* displayed the lowest stability, making it the least appropriate as an interior reference gene. The further RT-qPCR analysis on four prospect genes related to *L. discolor*’s development revealed that when *ACT* was used as the recommendation gene, the expression patterns followed transcriptome data. In contrast, utilizing *UBQ2* as the referral genetics led to expression patterns that diverged from the transcriptome data. This study notes the initial investigation right into the interior recommendation genetics of *L. discolor* and with the verification of genes connected with flower development, developing *ACT* as the most suitable interior referral genetics. These findings provide a trusted recommendation for succeeding gene expression evaluations in *L. discolor* and offer a basis for picking interior reference genes in carefully related species.

## Figures and Tables

**Table 1:** Primer sequences for internal control genes.

Gene ID	Primer Sequence (5′–3′)	Primer Sequence (3′–5′)	Amplicon Length/bp	Tem/°C	Blast Result	Gene
evm.model.tig36.167	GCTATGCTCGATGAGCCACT	GTTCTTTCCTCGCCAAGTGC	131	59.9 °C 59.7 °C	Protein phosphatase 2A	*PP2A*
evm.model.tig46.401	TGGCACAGGATCTGGATTGG	TTATACGGCTCCACGACTGC	129	59.7 °C 59.9 °C	β-tubulin1	*TUB*
evm.model.tig135.74	CTCCTGCTGGCATTAGTGGT	CTCGGTGAATTGCAGCGTC	125	59.7 °C 59.5 °C	Ubiquitin1	*UBQ1*
evm.model.tig38.70	AGCACGGAGCTCTTGATTCG	AAGCTAGCACAGCGTGACTC	106	60.4 °C 60.3 °C	Histone superfamily protein	*HIS*
evm.model.tig130.179	AGTGGACCAGGCAATCCTTG	GCAACTGAAGCCAGCACTTC	108	59.9 °C 60.0 °C	Ubiquitin1	*UBQ2*
evm.model.tig64.104	CCGTGCTTTCCCTTTATGCC	TGTGGGAGTGCATAGCCTTC	106	59.5 °C 59.7 °C	Actin1	*ACT*
evm.model.tig65.234	GAACCACCCTGGACAGATCG	AAGCTCCTTTCCAGATCGCC	121	60.1 °C 60.1 °C	Elongation factor 1-α	*EF1-α1*
evm.model.tig13.434	CTGTTGAGGATGTGCCCTGT	GGATGGGCATCAACCTCCTT	106	59.9 °C 59.7 °C	Elongation factor 1-α	*EF1-α2*
evm.model.tig14.338	GAGGTAGAGAGCAGCGACAC	TCCGACCATCTTCCAACTGC	118	59.9 °C 60.0 °C	Ubiquitin1	*UBQ3*

**Table 2:** Primer sequences for four genes of floral development.

Gene ID	Gene Name	Primer Sequence (5′–3′)	Primer Sequence (3′–5′)	Amplicon Product Length (bp)
evm.model.tig63.29	*LdMYB1*	GGGAGTGAACGAGACGGTTC	AGCTTGGAATGCCCGATCTC	135
evm.model.tig37.173	*LdMYB2*	GCAGCTCGTGGAAGAGTTTG	AATGGCCGCCTCTTGATTCT	129
evm.model.tig23.210	*LdMYB3*	TGCTTCCAAGCTCAAGCTCC	TAGGGGAAGAATCGGTGGGT	149
evm.model.tig6.584	*LdMYB4*	AGATCAGGGAAGAGTTGCCG	AAAGAGGCGAGCGATCACAG	147

**Table 3:** The amplification efficiency and correlation coefficient of nine candidate reference genes.

Gene	Amplification Efficiency (%)	Correlation Coefficient (R^2^)	Slope (K)
*EF1-α1*	108.41	0.995	−3.135
*ACT*	109.21	0.994	−3.119
*HIS*	98.29	0.997	−3.363
*TUB*	95.58	0.992	−3.432
*EF1-α2*	90.21	0.994	−3.581
*PP2A*	97.37	0.996	−3.386
*UBQ1*	101.67	0.989	−3.282
*UBQ3*	95.31	0.981	−3.439
*UBQ2*	93.68	0.999	−3.483

**Table 4:** Gene expression stability ranking of 9 candidate reference genes calculated by geNorm.

Ranking	Gene Name	Stability *M* Value
1	*ACT/HIS*	0.545
2	*EF1-α1*	0.632
3	*TUB*	0.773
4	*EF1-α2*	0.888
5	*UBQ1*	0.963
6	*PP2A*	1.084
7	*UBQ3*	1.243
8	*UBQ2*	1.341

**Table 5:** Gene expression stability ranking of 9 candidate reference genes calculated by NormFinder.

Ranking	Gene Name	Stability Value
1	*EF1-α1*	0.506
2	*ACT*	0.592
3	*HIS*	0.7
4	*TUB*	0.749
5	*EF1-α2*	0.938
6	*PP2A*	1.098
7	*UBQ1*	1.134
8	*UBQ3*	1.332
9	*UBQ2*	1.459

**Table 6:** Gene expression stability ranking of 9 candidate reference genes calculated by BestKeeper.

Ranking	Gene Name	SD	CV
1	*PP2A*	0.36	1.53
2	*UBQ3*	0.82	3.75
3	*ACT*	0.85	4.26
4	*EF1-α1*	0.93	4.80
5	*TUB*	0.94	4.00
6	*UBQ2*	0.99	4.57
7	*HIS*	1.15	5.11
8	*EF1-α2*	1.20	5.71
9	*UBQ1*	1.55	7.34

**Table 7:** Gene expression stability ranking of 9 candidate reference genes calculated by ΔCt method.

Ranking	Gene Name	Stability Value
1	*EF1-α2*	1.09
2	*ACT*	1.12
3	*HIS*	1.15
4	*TUB*	1.22
5	*EF1-α2*	1.32
6	*UBQ1*	1.43
7	*PP2A*	1.44
8	*UBQ3*	1.60
9	*UBQ2*	1.69

**Table 8:** Gene expression stability ranking of 9 candidate reference genes calculated by comprehensive analysis.

Ranking	Gene Name	Geometric Mean Value
1	*ACT*	1.86
2	*EF1-α1*	1.86
3	*HIS*	2.82
4	*PP2A*	4.14
5	*TUB*	4.23
6	*EF1-α2*	5.62
7	*UBQ3*	5.66
8	*UBQ1*	6.90
9	*UBQ2*	8.13

## Data Availability

Data are contained within the article.
